# Supplementary material for: Winnie-APCMin/+ Mice: A Spontaneous Model of Colitis-Associated Colorectal Cancer Combining Genetics and Inflammation
Source: Int J Mol Sci. 2020 Apr 23;21(8):2972. doi: 10.3390/ijms21082972 (PMC7215554; doi:10.3390/ijms21082972)
Supplement: Supplementary file 1 [file ijms-21-02972-s001.pdf]

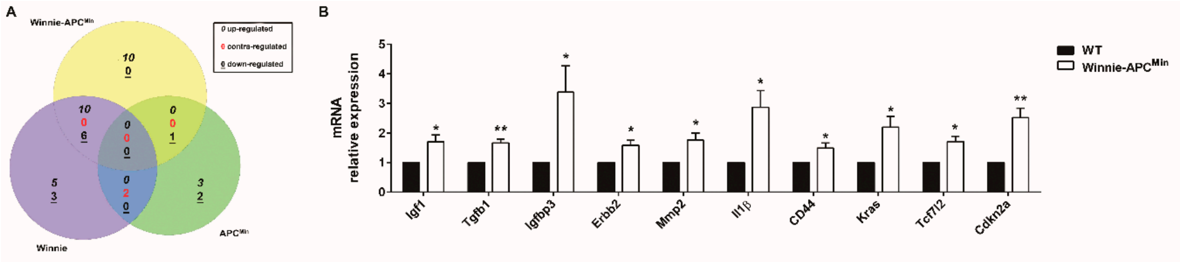

Figure S1. Molecular characterization of 5-wk-old Winnie-*APC*$^{Min/+}$ distal colon. (**a**) Venn diagram reported qPCR data obtained comparing Winnie-*APC*$^{Min/+}$, Winnie and *APC*$^{Min/+}$ relative to WT mice. (**b**) Histogram indicates relative expression of 10 specifically up-regulated genes in Winnie-*APC*$^{Min/+}$ (white bars) versus WT mice (black bars) (n=4 animals/group). Histogram represents the mean ± SEM. *p<0.05, **p<0.01.

Table S1. Fertility data relative to the breeding strategy of Winnie and Winnie-*APC*$^{Min/+}$ mice.

Breeders Genotype	Breeding pairs (n)	Litters (n) ±SD	Mean Litter Size
M Winnie$^{+/-}$ x F Winnie$^{+/-}$	23	54 ± 3.3	6
M Winnie$^{+/-}$ *APC*$^{Min/+}$ x F Winnie$^{+/-}$	31	40 ± 1.3	5.2
